# Interplay Between Immune and Cancer-Associated Fibroblasts: A Path to Target Metalloproteinases in Penile Cancer

**DOI:** 10.3389/fonc.2022.935093

**Published:** 2022-07-19

**Authors:** Sarah Santiloni Cury, Hellen Kuasne, Jeferson dos Santos Souza, Juan Jose Moyano Muñoz, Jeyson Pereira da Silva, Ademar Lopes, Cristovam Scapulatempo-Neto, Eliney Ferreira Faria, Jean-Marie Delaissé, Fabio Albuquerque Marchi, Silvia Regina Rogatto

**Affiliations:** ^1^ Department of Clinical Genetics, University Hospital of Southern Denmark, Vejle, Denmark; ^2^ Institute of Regional Health Research, University of Southern Denmark, Odense, Denmark; ^3^ Department of Structural and Functional Biology, São Paulo State University (UNESP), Botucatu, Brazil; ^4^ Rosalind and Morris Goodman Cancer Institute, McGill University, Montreal, QC, Canada; ^5^ International Research Center (CIPE), A. C. Camargo Cancer Center, São Paulo, Brazil; ^6^ Universidad Señor de Sipán, Chiclayo, Peru; ^7^ Pelvic Surgery Department, A. C. Camargo Cancer Center, São Paulo, Brazil; ^8^ Molecular Oncology Research Center, Barretos Cancer Hospital, Barretos, Brazil; ^9^ Department of Pathology, Diagnósticos da América - DASA, Barueri, Brazil; ^10^ Uro-oncology and Robotic Surgery, Hospital Felicio Rocho, Belo Horizonte, Brazil; ^11^ Clinical Cell Biology, Lillebaelt Hospital, University Hospital of Southern Denmark, Vejle, Denmark; ^12^ Department of Clinical Research, Clinical Cell Biology, University of Southern Denmark, Odense, Denmark

**Keywords:** penile cancer, secretome, transcriptome, cancer-associated fibroblasts, response to therapy

## Abstract

Extracellular matrix (ECM) remodeling and inflammation have been reported in penile carcinomas (PeCa). However, the cell types and cellular crosstalk involved in PeCa are unexplored. We aimed to characterize the complexity of cells and pathways involved in the tumor microenvironment (TME) in PeCa and propose target molecules associated with the TME. We first investigated the prognostic impact of cell types with a secretory profile to identify drug targets that modulate TME-enriched cells. The secretome analysis using the PeCa transcriptome revealed the enrichment of inflammation and extracellular matrix pathways. Twenty-three secreted factors were upregulated, mainly collagens and matrix metalloproteinases (MMPs). The deregulation of collagens and MMPs was confirmed by Quantitative reverse transcription - polymerase chain reaction (RT-qPCR). Further, the deconvolution method (digital cytometry) of the bulk samples revealed a high proportion of macrophages and dendritic cells (DCs) and B cells. Increased DCs and B cells were associated with better survival. A high proportion of cancer-associated fibroblasts (CAFs) was observed in low-survival patients. Patients with increased CAFs had decreased immune cell proportions. The treatment with the MMP inhibitor GM6001 in CAF cells derived from PeCa resulted in altered cell viability. We reported a crosstalk between immune cells and CAFs, and the proportion of these cell populations was associated with prognosis. We demonstrate that a drug targeting MMPs modulates CAFs, expanding the therapeutic options of PeCa.

## Introduction

Penile cancer (PeCa) represents 0.2% of all cancers diagnosed worldwide (1). However, poor and developing countries have a high incidence of the disease (2–4). Partial penectomy is frequently used for localized carcinomas (5). The disease could be aggressive, metastatic, and mutilating, mainly due to the delay in seeking treatment (6). Despite all efforts to improve the therapeutic strategies, the survival rates of PeCa patients remained almost unchanged over the past years (7).

Molecular and functional studies have revealed an important role of cells composing the tumor microenvironment (TME) in PeCa. The presence and distribution of immune checkpoint molecules or immune cell components were shown to be a potential predictor of clinical outcomes [reviewed in Aydin et al. (8)]. However, the immune fraction of TME alone is insufficient to predict treatment response and survival (8). Cancer-associated fibroblasts (CAFs) are a key component of the TME, playing a critical role in the extracellular matrix (ECM) deposition and remodeling. Moreover, CAFs have been implicated in the modulation of the immune system by establishing an immunosuppressive stroma, which can promote resistance to immune-based therapies (9). Although a limited number of transcriptome analyses in PeCa has been reported (7), the enrichment of pathways associated with ECM organization was described in patients with lymph node (LN) metastasis (10).

Immunotherapeutic drugs inhibit the immune checkpoint genes such as programmed cell death 1 (PD-1) and its ligand (PD-L1) (11). Cocks et al. (2016) identified PD-L1 expression in approximately 40% of PeCa patients, who may benefit from immunotherapies (12). Immunotherapy was further supported by studies that found that most patients presented advanced cancer (12, 13). The remaining 60% of PeCa patients have limited therapeutic options, including organ amputation and standard-of-care chemotherapies. In these cases, the immunotherapy response could be enhanced using a combinatorial treatment with TME-modulating drugs (14). Targetable molecular mechanisms that modulate CAFs are suggested to increase the cytotoxic T-cell level in the tumor, contributing to an increased immunotherapy response (15). Despite efforts to characterize the immune environment in PeCa (8, 13, 16, 17), there is a lack of in-depth knowledge on how the immune cells and CAFs simultaneously affect tumor progression.

The transcriptome analysis of bulk tumor samples allows *in silico* deconvolution using computational tools to infer cell type proportions (18). Moreover, the tumor transcriptome profile of the secretome (genes encoding secreted proteins) indicates which cell is activated in the tumor and releases factors that allow communication with other cells (19–21). These strategies are valuable tools to identify enriched cell types within the TME and their contribution to the tumor progression and response to therapy.

Here, we explored the transcriptome from two perspectives: 1) identify enriched immune and stromal cells using an *in silico* deconvolution method and 2) investigate targetable secretome components for TME modulation in PeCa. These strategies allowed us to characterize the TME composition of PeCa, in which we verified an enrichment of CAFs inversely correlated with immune cell proportion and an association with poor survival. Once the TME was characterized, the next step was to evaluate genes associated with ECM remodeling to identify potential drug targets able to modulate CAFs. Among these genes, we confirmed high expression levels of matrix metalloproteinase (MMP) genes in PeCa samples. Using PeCa–derived CAFs, we inhibited MMP expression and demonstrated a low viability of the cells.

## Materials and Methods

### Patients and Samples

A cohort of 63 squamous cell penile carcinomas (PeCa) usual type, 16 adjacent normal tissues, and 13 histologically normal glands (obtained from necropsies) from patients treated at A.C.Camargo Cancer Center and Barretos Cancer Hospital, São Paulo, Brazil, from 2006 to 2015 were included in the present study (**Table S1**). The entire cohort of 63 patients was distributed as described in the flowchart (**Figure S1**). The Human Research Ethics Committee from both Institutions approved the study (Protocols 1884/14 and 1030/2015, respectively). All patients and or family members were informed regarding the protocols and provided written informed consent before sample collection. The study was conducted according to the guidelines of the Declaration of Helsinki. The human papillomavirus (HPV) genotyping was performed using the Linear Array HPV Test Genotyping (Roche Molecular Diagnostics, Branchburg, NJ, USA).

### Transcriptomic Analysis

Transcriptomic analysis was performed in 16 PeCa compared to six histologically normal penile glans using the GeneChip™ Human Transcriptome Array 2.0 (HTA 2.0; Affymetrix Santa Clara, California, USA), following the manufacturer’s recommendations. The HTA 2.0 platform (Affymetrix, USA) was designed to interrogate >6 million probes targeting coding and non-coding transcripts, and exon–exon splice junctions (245,349 protein coding transcripts and 40,914 non-coding transcripts). RNA was isolated from fresh-frozen samples using the RNeasy mini kit (Qiagen, Valencia Germantown, Maryland, USA). RNA integrity was verified in all samples using the Agilent 2100 Bioanalyzer RNA 6000 LabChip kit (Agilent Technologies, Santa Clara, CA, USA). The scanning was performed using Affymetrix GeneChip Scanner 7000 (Affymetrix/ThermoFisher Waltham, Massachusetts, USA). The CEL files were generated by Affymetrix^®^ GeneChip^®^ Command Console^®^ (AGCC) 4.0. The Transcriptome Analysis Console (TAC, ThermoFisher, USA, v.4.0) was used for data normalization and differential expression analysis. Microarray data are available on the Gene Expression Omnibus (GEO) database (GSE196978). We also explored the transcriptomic profile of 30 usual PeCa previously evaluated by our group (Whole Human Genome Microarray 4x44K; Agilent Palo Alto, California, USA) (GSE57955) (22). Two datasets were analyzed independently (human GRCh37/hg19 annotation). The differentially expressed genes (DEGs) from the internal dataset were selected considering |fold change (FC)|> 2 and FDR <0.01. For the Agilent microarray data, DEGs were selected when presenting a log2 Cy3/Cy5 mean ratio ≥1.0 or ≤1.0 within a 99% confidence interval (CI) (upregulated and downregulated, respectively).

### Transcriptome-Based Secretome Analysis

The upregulated genes identified in PeCa samples from each platform (Affymetrix and Agilent) were selected for secretome analysis using The Human Protein Atlas (HPA) database (www.proteinatlas.org) (23) with 2,943 predicted secretome proteins. The secretome genes were visualized using the protein–protein interaction (PPI) network with the Search Tool for the Retrieval of Interacting Genes/Proteins (STRING) tool v.11.5 (24) (http://string-db.org/). We considered experiments, database, co-expression, and co-occurrence as active interaction sources. The minimum required interaction score was 0.9 (highest confidence), and the disconnected nodes in the network were hidden for display simplification. The PPI p-values <0.05 were considered significant. The visualization and data annotation of PPI networks were constructed using Cytoscape v3.8.2.

Functional enrichment analysis was performed using the Enrichr tool (https://maayanlab.cloud/Enrichr/) (25) by accessing the libraries Gene Ontology (GO) biological process, GO Cellular Component, GO Molecular Function, Kyoto Encyclopedia of Genes and Genomes (KEGG), MSigDB Hallmark, Reactome, and Wiki Pathways. The terms were enriched with adjusted p-values <0.001. Ingenuity Pathway Analysis (IPA) software was used to identify molecules that potentially target the secretome genes.

### Gene Expression Analysis by Real-Time Quantitative Polymerase Chain Reaction

The gene expression levels of matrix metalloproteinase (MMP) genes (*MMP1*, *MMP3*, *MMP7*, *MMP9*, *MMP10*, *MMP12*, and *MMP13*) and collagens (*COL1A2*, *COL3A1*, *COL4A1*, *COL5A2*, *COL10A1*, *COL11A1*, and *COL24A1*) were investigated in 47 PeCa aiming to confirm the transcriptomic results. Primer sets were designed using Primer-Blast software (http://www.ncbi.nlm.nih.gov/tools/primer-blast/) (**Table S2**). Total RNA was converted into complementary DNA (cDNA), and the amplification was carried out as previously described (10). We used *GUSB* as reference transcript (26). The relative quantification of mRNA expression was evaluated using the 2^−ΔΔCT^ method (27). Data were analyzed statistically using Graphpad Prism 5.0 (GraphPad Software Inc., La Jolla, CA, USA). The Mann–Whitney U test was used to compare normal vs. cancer groups. *P*-values < 0.05 were considered significant.

### Immune Score Classification

The transcriptome deconvolution analysis was performed in the internal set of samples to evaluate the prevalence of immune infiltrating cells. The digital cytometry analysis was conducted using the CIBERSORTx tool (https://cibersortx.stanford.edu/) to impute the immune cell fractions of 22 cell types (LM22 matrix signature) from the bulk RNA-seq data (28). We applied the default settings of CIBERSORTx and batch correction to minimize the impact of cross-platform variation. The immune scores (CIBERSORTx) were used to classify the PeCa samples as “immune hot” high immune cells infiltration and “immune cold” low immune cells infiltration (29). The immune score cut-offs for macrophages, DCs, and B cells associated with survival were also determined (EasyROC v. 1.3.1, http://www.biosoft.hacettepe.edu.tr/easyROC/) (30).

### Cancer-Associated Fibroblast Score

The EPIC (http://epic.gfellerlab.org/) tool was used to estimate the fraction of CAFs and explore the changes in the matrix components of PeCa and normal tissues (internal set) (31). EPIC establishes reference gene expression profiles for major tumor-invasive immune cell types (CD4+ T, CD8+ T, B, natural killer, and macrophages) and further deduces the reference spectra of CAFs and endothelial cells (32).

The digital cytometry analysis (CIBERSORTx tool) was applied to impute the CAF proportion in PeCa samples using the CAF expression signature from single-cell RNA-seq data from head and neck squamous cell carcinomas (HNSCC) (33). First, the CAF signature matrix and CAF fractions were imputed in PeCa (default settings and batch correction). We used the CAF signature derived from HNSCC due to the absence of single-cell resolution data in PeCa samples. The criteria to select HNSCC as a reference for CAFs were based on similarities shared by these tumors, including that both are derived from epithelial cells, they are classified as squamous cell carcinomas (34), and HPV is an etiological factor–associated disease (35). In addition to CAFs from HNSCC (36), a consensus list of canonical CAF markers of human cancers was obtained (9, 37–39). The expression signature of CAF markers was compared with CAF classification using digital cytometry to confirm the reliability of the CAF signature in PeCa.

### Cancer-Associated Fibroblasts Derived From Penile Cancer Cells

In a previous study, we established three cells derived from PeCa (Cell4, Cell5, and Cell6) that were molecularly and morphologically characterized as CAFs (40). The morphology of CAF in PeCa was evaluated by immunofluorescence using Texas Red: actin/phalloidin (Thermo Fisher Scientific, Waltham, MA, USA), FITC (fluorescein isothiocyanate): tubulin (Thermo Fisher Scientific, Waltham, MA, USA), and DAPI (4′,6-diamidino-2-phenylindole): nucleus (Vector Laboratories, Burlingame, CA, USA) as described by Kuasne et al. (40).

We performed chemosensitivity assays using GM6001 (Merck Life Science, Hellerup, Denmark), a broad-spectrum MMP inhibitor (MMP-1, MMP-2, MMP-3, MMP-7, MMP-8, MMP-9, MMP-12, MMP-14, and MMP-26). Briefly, PeCa cells were seeded in a 96-well plate at a density of 1 × 10^5^ cells/ml and incubated at 37°C in a complete medium composed of 3:1 keratinocyte serum-free medium–DMEM/F12 (Dulbecco’s modified Eagle medium/nutrient mixture F-12) (GIBCO, Carlsbad, CA, USA) supplemented following the previously described protocol (40). Treatment with GM6001 was administered after 24 h in concentrations of 0, 1, 3, 10, and 20 μM, and six replicates were used for each concentration. Following 24 h of treatment incubation, 100 μl of MTT (3-(4,5-dimethylthiazol-2-yl)-2,5-diphenyltetrazolium bromide) reagent solution (0.5 mg/ml) was added to each well and incubated for 3 h at 37°C. After removing the MTT solution, 180 μl of DMSO (dimethyl sulfoxide) was added to solubilize the violet formazan crystals. The plates were incubated for 15 min at 37°C, and the absorbance readings were performed at 560 nm with a reference of 690 nm using the Biotek Synergy HT microplate reader (Agilent, Santa Clara, CA, USA).

### Data Representation and Statistical Analyses

Heatmaps were created using the web tool Morpheus (https://software.broadinstitute.org/morpheus). GraphPad Prism^®^ (GraphPad Software, v5.0, 2008, USA) was used for statistical analysis. Log-rank (Mantel–Cox)–Gehan–Breslow–Wilcoxon Tests were used for survival analysis.

## Results

The patients included in this study showed similar clinical and histopathological characteristics, such as mean age, alcohol consumption, tobacco usage, HPV status, TNM stage, and perineural and angiolymphatic invasion (**Table S1**).

**Figure 1:**
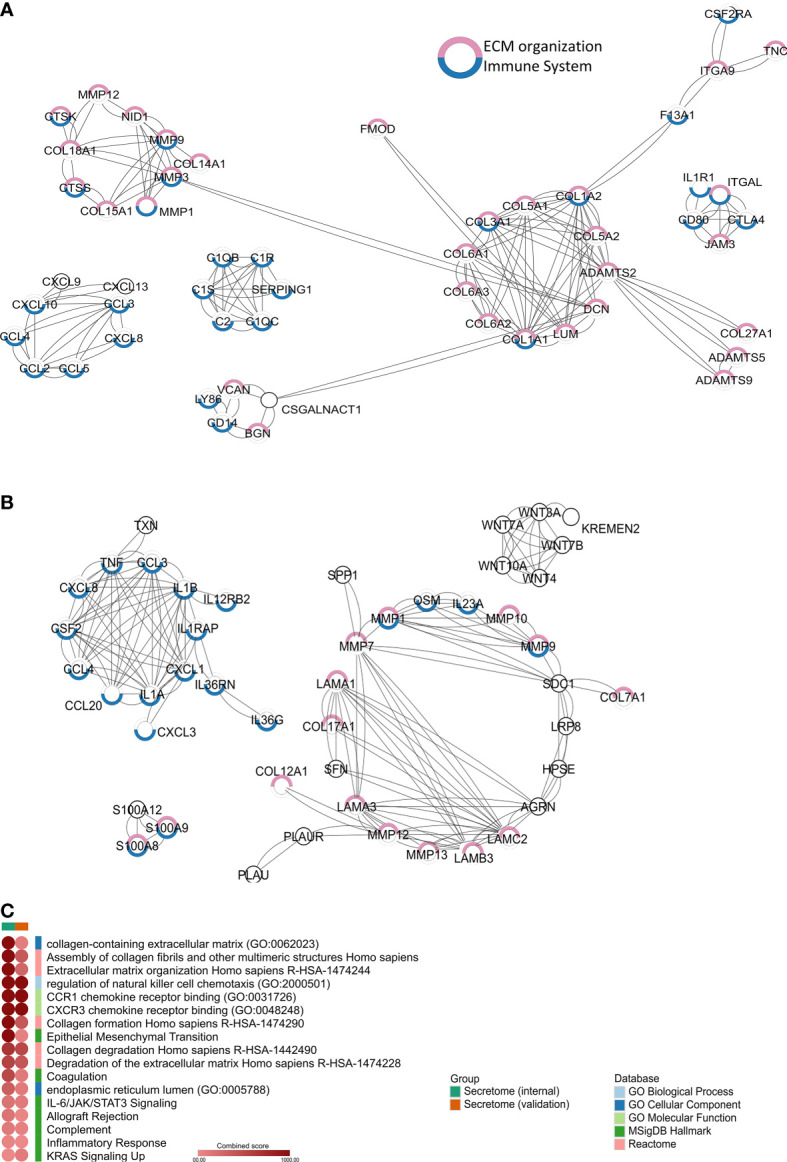
Secretome profile of penile cancer (PeCa) **(A)** Protein–protein interactions (PPIs) of secretome genes upregulated in PeCa from the internal dataset (Affymetrix). **(B)** PPIs of secretome genes upregulated in PeCa from the validation dataset (Agilent). Network generated by STRING (Search Tool for the Retrieval of Interacting Genes/Proteins) using the highest confidence interaction score (0.9). Colored circles indicate the associated ontology; genes associated with the immune system and extracellular matrix (ECM) are highlighted in blue and pink, respectively. Edges represent interaction. **(C)** Heat-scatter plot of the combined score for the enriched pathways and ontologies. Top categories selected from enrichment analysis of secretome genes from PeCa samples. The intensity of the color in the dotplot indicates the enrichment significance by the combined score. Significant adjusted *p*-value was found in all included terms. Gene set names are colored according to the Gene Ontology (GO) biological process (light blue), GO cellular component (dark blue), GO molecular function (light green), Kyoto Encyclopedia of Genes and Genomes (KEGG, dark green), MSigDB Hallmark (pink), Reactome (red), and Wiki Pathways (orange).

We identified 2,199 and 1,050 upregulated genes in PeCa compared with normal tissues in our internal and validation datasets, respectively, of which 161 and 189, respectively, were predicted to encode secreted proteins. The PPI analysis of the secretome genes revealed functions associated with ECM and inflammation (**Figures 1A, B**). Seventeen terms with the highest combined score were mainly associated with inflammatory response and ECM regulation in both PeCa datasets (**Table S3**; **Figure 1C**). Despite enriching similar pathways and ontologies, only 23 secretory genes (encoding inflammatory cytokines/chemokines and ECM molecules) were upregulated in internal and validation datasets (**Table 1**). These findings suggested that PeCa cells directly interact with the immune system and the stroma.

**Table 1:** Twenty-three genes encoding for secreted proteins upregulated in internal (n=16) and validation (n=30) datasets of penile cancer.

Gene Symbol	Gene Name	Function*
*ADAMDEC1*	ADAM Like Decysin 1	Immune response and metalloendopeptidase activity
*CCL3*	C-C motif chemokine 3	Inflammatory response
*CCL4*	C-C motif chemokine 4	Inflammatory response
*CEMIP*	Cell migration–inducing and hyaluronan-binding protein	Regulates epithelial–mesenchymal transition
*COL7A1*	Collagen alpha-1(VII) chain	Extracellular matrix structure
*CXCL13*	C-X-C motif chemokine 13	Inflammatory response
*CXCL8*	C-X-C motif chemokine 8	Inflammatory response
*CXCL9*	C-X-C motif chemokine 9	Inflammatory response
*EGFL6*	Epidermal growth factor–like protein 6	Extracellular matrix organization
*ESM1*	Endothelial cell–specific molecule 1	Angiogenesis
*FABP5*	Fatty acid–binding protein 5	Lipid metabolism
*GZMA*	Granzyme A	Immune response
*ICOS*	Inducible T-cell costimulator	Immune response
*LGALS9*	Galectin-9	Inflammatory response
*MMP1*	Interstitial collagenase	Extracellular matrix degradation
*MMP12*	Macrophage metalloelastase	Extracellular matrix degradation
*MMP9*	Matrix metalloproteinase-9	Extracellular matrix degradation
*PGLYRP4*	Peptidoglycan recognition protein 4	Immune response
*PI3*	Elafin	Immune response
*PLA2G7*	Platelet-activating factor acetylhydrolase	Lipid metabolism
*S100A7*	S100-A7	Immune response
*S100A8*	S100-A8	Immune response
*S100A9*	S100-A9	Immune response

^*^Information retrieved from Uniprot database – UniProtKB 2021_04 (https://www.uniprot.org/, Accessed November 2021).

### Tumor Microenvironment Immune Composition of Penile Cancer

**Figure 2:**
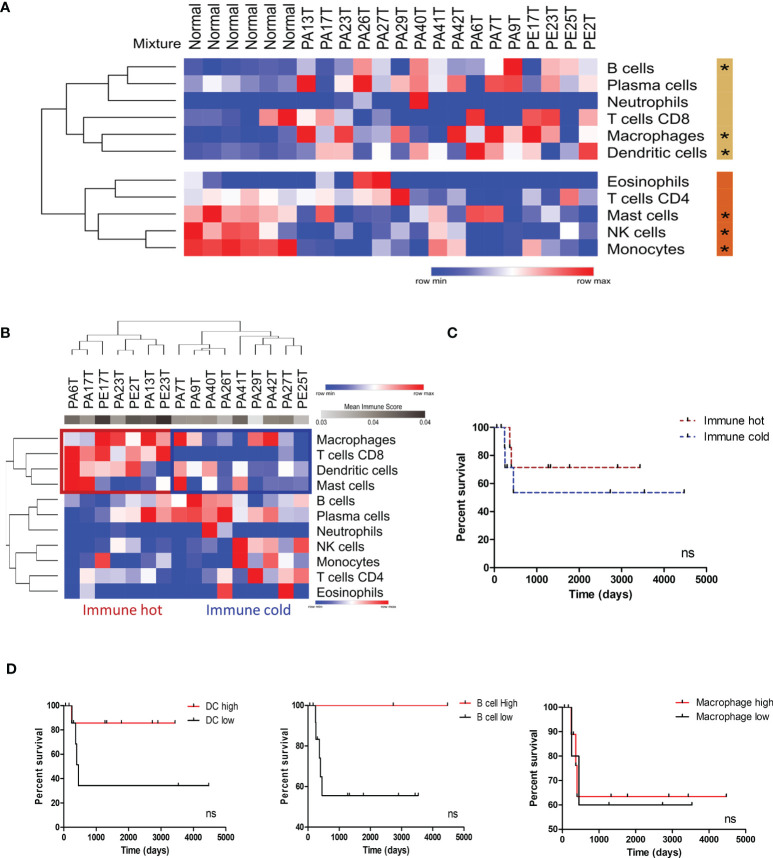
Immune profile characterization of PeCa samples using digital cytometry. **(A)** Heatmap representative of the immune cell score in normal and PeCa samples calculated using CIBERSORTx. (*) significant p-values comparing tumor versus normal samples. Rows were clustered based on the Euclidean distance of immune score values. Two clusters were generated using K-means analysis (K-means = 2). The beige and orange bars indicate the clusters of cells enriched in PeCa samples and normal samples, respectively. **(B)** Heatmap representative of immune cell scores in PeCa samples calculated using CIBERSORTx. Rows and columns were clustered based on the one minus Pearson correlation of immune score values. **(C)** Kaplan–Meier plot of immune hot and immune cold PeCa patients based on Figure 2B. **(D)** Kaplan–Meier plot of patients presenting high and low scores of dendritic cells (DCs), B cells, and macrophages. The bets cutoffs for survival analysis were determined by the easyROC web tool. **(C, D)** The Gehan–Breslow–Wilcoxon Test determined the hazard ratio (HR) with 95% confidence intervals (CIs). ns: p-values not statistically significant.

Based on the significance of inflammation-associated pathways and the immune system–related genes in the PeCa secretome (**Figure 1**; **Table 1**), we first identified enriched immune cells within the TME using an *in silico* deconvolution by applying digital cytometry. PeCa samples presented a higher proportion of dendritic cells (DCs), macrophages, and B cells, while normal samples presented a high number of monocytes, NK cells, and mast cells (**Figure 2A**). We identified a set of PeCa patients with high scores of CD8 T cells, macrophages, and DCs and higher mean immune score (immune hot; **Figure 2B**). Although not significant, immune-cold patients had a trend to present shorter overall survival (**Figure 2C**). Since we found an increased proportion of macrophages, DCs, and B cells in PeCa compared to normal samples and differential scores among the tumor samples, we next investigated the association of these cells with overall survival. The best score cutoff for macrophage, DC, and B cells was calculated using the easyROC tool (30). The optimal immune score cutoff generated was 0.023 for macrophages, 0.059 for DCs, and 0.093 for B cells. Values above these cutoffs were considered as high. Patients with higher DC and B cell scores also had a trend toward higher overall survival (**Figures 2C, D**).

### Cancer-Associated Fibroblast Profile

**Figure 3:**
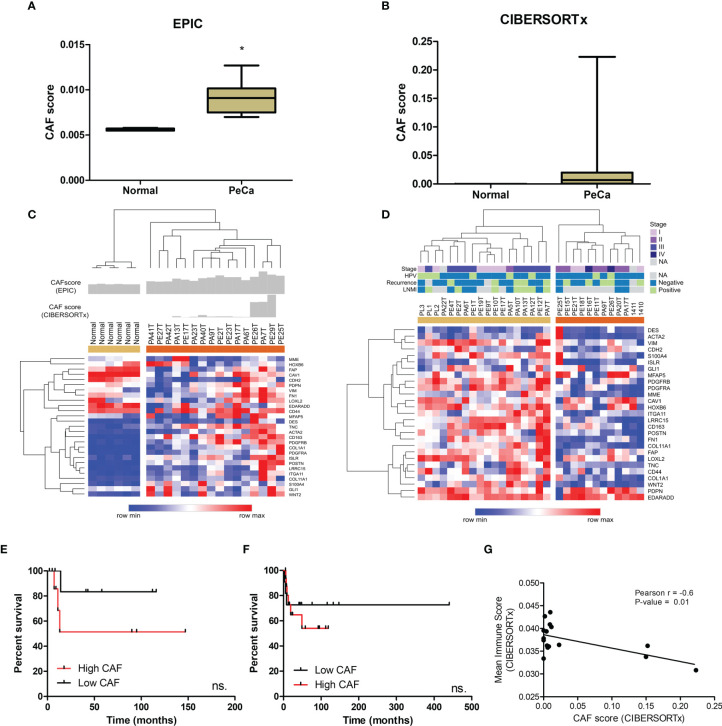
CAF characterization of PeCa samples using digital cytometry. **(A)** Bar graph demonstrating the mean score estimated using EPIC. The statistical significance was analyzed using Student’s t-test. *P < 0.001. **(B)** Bar graph demonstrating mean score estimated using CIBERSORTx. **(C)** Heatmap representing the gene expression of CAF markers in the internal set of cases (Affymetrix). The top panel indicates the CAF score in normal and PeCa samples calculated using CIBERSORTx and EPIC. Rows and columns were clustered based on the Euclidean distance of CAF marker expression. Three clusters were generated using k-means analysis (K-means = 3). **(D)** Heatmap representing the gene expression of CAF markers in the validation dataset (Agilent). Rows and columns were clustered based on Euclidean distance of CAFs marker expression. Two clusters were generated using k-means analysis (K-means = 2). **(E)** Kaplan–Meier plot of patients presenting high and low scores of CAFs (Affymetrix; internal set). **(F)** Kaplan–Meier plot of patients presenting high and low expression of CAF markers (Agilent; validation set). **(E, F)** The HR with 95% confidence intervals (CI) was determined by the Gehan–Breslow–Wilcoxon Test. ns, not statistically significant. **(G)** The partial Pearson’s rank correlation (r) and p-value are given for the CAF score generated by CIBESORTx with the mean immune score also generated by CIBESORTx.

Since we identified enriched pathways associated with ECM organization, the next step was to assess the presence of CAFs possibly involved in the synthesis of ECM remodeling factors. We also investigated a potential interplay between immune cells and CAFs. The CAF score (EPIC tool) in tumor samples was higher and statistically significant (p<0.0001) compared to normal tissues (**Figure 3A**). To deconvolute the CAFs from PeCa and normal samples (CIBERSORTx), we used a gene signature of CAFs derived from the HNSCC single-cell RNA-seq study (33). We found higher scores of CAFs in PeCa compared to normal samples (**Figure 3B**). Next, we analyzed the gene expression levels of 31 canonical CAF markers (*ACTA2, S100A4, VIM, DES, FAP, PDGFRB, CAV1, MME, GPR77, TNC, GLI1, HOXB6, LRRC15, Ly6c1, ISLR, PDGFRA, PDPN, MFAP5, COL11A1, ITGA11, NG2, POSTN, COL1A1, CDH2, FN1, CD44, CD90, CD163, LOXL2, EDARADD*, and *WNT2*) (9, 36–39). Interestingly, this signature was able to cluster PeCa (**Figure 3C**). Based on the CAF scores and gene expression, we noted a heterogeneous profile, where 31% of PeCa samples (PA41T, PE27T, PA42T, PA13T, and PE17T) presented a low expression of CAF markers (**Figure 3C**). We also found that cases with higher CAF scores and an increased expression of CAF markers presented low overall survival (**Figure 3E**). The PeCa samples from the validation dataset showed a cluster composed of 18 patients presenting a higher expression of CAF markers (beige cluster), while 12 patients (40%) showed low expression (orange cluster) (**Figure 3D**). Moreover, a potential association of CAF signature expression with survival was confirmed (**Figure 3F**).

A significantly negative correlation was found between the CAF score with the mean immune score (the mean score of all immune cell types calculated for each sample) (**Figure 3G**; **Table S4**).

### Genes Related to Extracellular Matrix Are Associated With Penile Cancer Development and Poor Outcome Features

**Figure 4:**
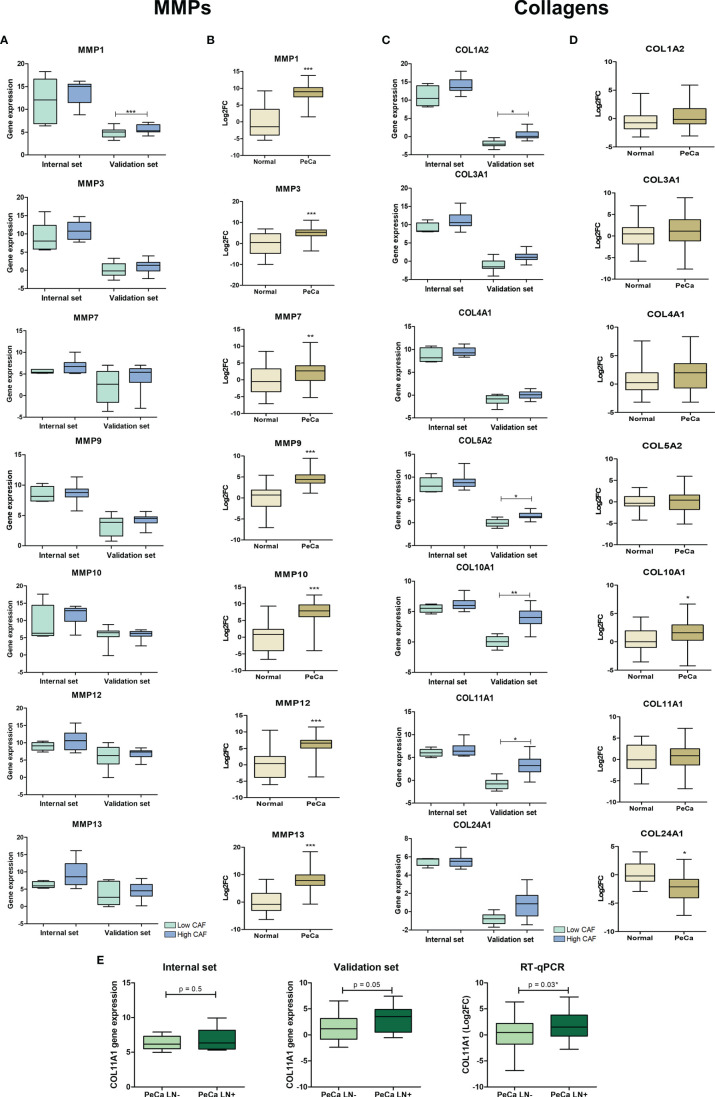
Expression pattern of matrix metalloproteinases and collagens in PeCa samples. **(A)** Box plots representative of expression levels of *MMP1*, *MMP3*, *MMP9*, *MMP10*, *MMP12*, and *MMP13* genes in PeCa compared to normal samples from internal [normalized-expression Robust Multi-ArrayAverage (RMA)] and validation set (expression ratio) according to the CAF score. **(B)** Box plots showing the expression levels of *MMP1*, *MMP3*, *MMP9*, *MMP10*, *MMP12*, and *MMP13* in PeCa samples compared to normal tissues using RT-qPCR [log_2_fold change (2−DDCt) relative to *GUSB*]. The statistical difference was analyzed by the Mann–Whitney U test. **(C)** Box plot representative of the expression levels of *COL11A1, COL1A2, COL4A1, COL3A1, COL5A2, COL10A1*, and *COL24A1* genes in PeCa samples from internal (normalized-expression RMA) and validation set (expression ratio) according to the CAF score. **(D)** Box plots showing the expression levels of *COL11A1, COL1A2, COL4A1, COL3A1, COL5A2, COL10A1*, and *COL24A1* genes in PeCa compared to normal samples using RT-qPCR [log_2_fold change (2−DDCt) relative to *GUSB*]. Statistical difference was analyzed by the Mann–Whitney U test. **(E)** Box plot showing the expression levels of *COL11A1* in PeCa compared to normal tissues from internal (normalized-expression RMA), validation set (expression ratio), and RT-qPCR according to lymph node (LN) metastasis. LN+: patients positive for LN metastasis; LN-: patients negative for LN metastasis. Statistical difference was analyzed by Student’s t-test. *p-values < 0.05, **p-values < 0.01, and ***p-values < 0.001.

The secretome of two datasets showed the enrichment of ontologies and pathways associated with ECM organization and degradation. Considering the interaction between collagen and MMPs (41), we evaluated their expression pattern on PeCa according to the CAF score and compared them to normal tissues. Our interest was also confirming the altered expression of these genes, and if confirmed, we investigated whether ECM proteins are targetable for TME modulation in PeCa. *MMP1* was the only metallopeptidase with significantly increased expression levels in PeCa with a high CAF score (validation set). However, most MMPs tended to increase expression in high CAF scores in PeCa (**Figure 4A**). A high expression of *COL11A1*, *COL1A2*, *COL5A2*, and *COL10A1* was detected in PeCa samples with high CAF scores (validation set, **Figure 4C**). Using RT-qPCR in a larger set of cases, we found that all MMPs tested presented increased expression in PeCa (**Figure 4B**). In addition, *COL10A1* showed significantly increased expression, and *COL24A1* showed down expression in PeCa compared to normal samples (**Figure 4D**). A significantly increased *COL11A1* expression was found in patients with LN involvement (RT-qPCR) and presented a trend toward significance in microarray datasets (**Figure 4E**).

### Cancer-Associated Fibroblasts Derived From Penile Cancer Cells Are Sensitive to Matrix Metalloproteinase Inhibitor

**Figure 5:**
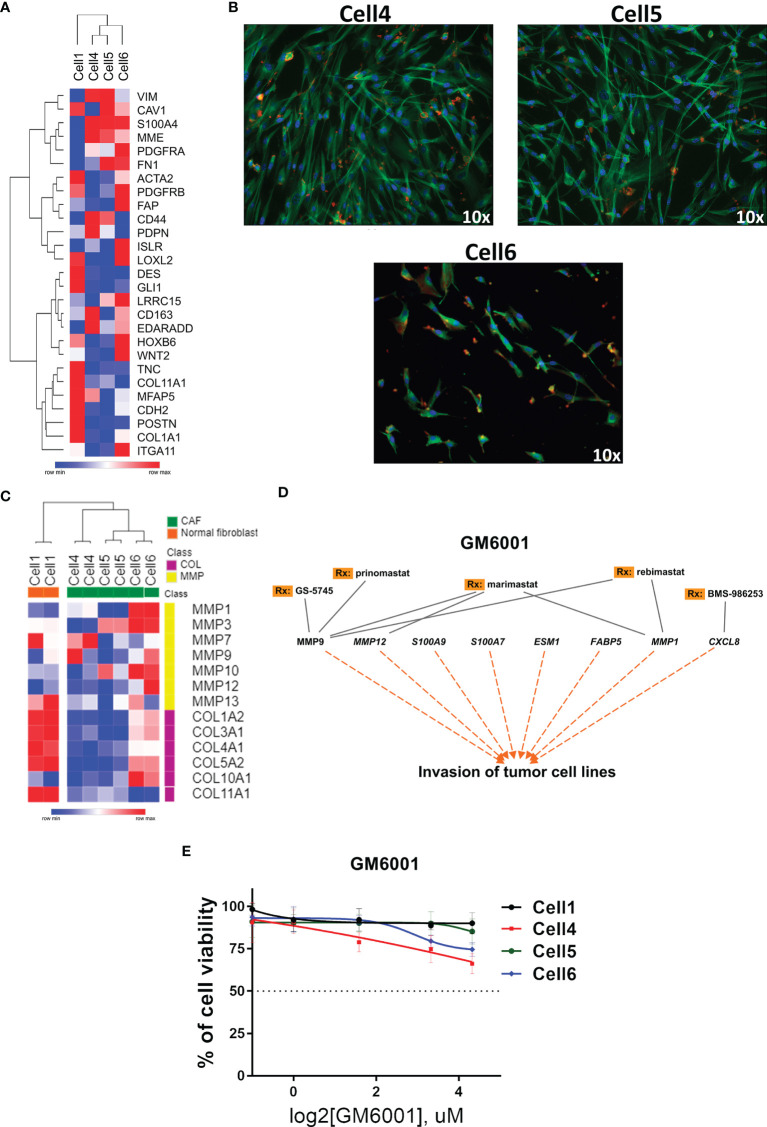
Targeted therapy in PeCa–derived CAF cells. **(A)** Heatmap representative of gene expression of CAF markers in PeCa–derived cells (Cell4, Cell5, and Cell6) and normal foreskin cell line (Cell 1). Rows and columns were clustered based on the Euclidean distance of CAF marker expression. **(B)** Immunofluorescence images (Texas Red: actin/phalloidin; FITC: tubulin; and DAPI: nucleus, ×10 magnification, Nikon TE2000) of CAF cells (Cell4, Cell5, and Cell6). **(C)** Heatmap representative of the expression levels of MMP and collagen genes (same gene set used in the validation) in PeCa-derived cells (Cell4, Cell5, and Cell6) and Cell1. **(D)** Potential target therapy for secreted genes, especially MMPs (IPA analysis). **(E)** Cell viability assay using an MMP inhibitor (GM6001—Pan inhibitor of MMPs) at the indicated concentrations for 24 h to treat Cell1, Cell4, Cell5, and Cell6.

Considering the global alteration of MMPs in PeCa and their potential to promote an immunosuppressive TME by remodeling it (42), we investigated the expression of CAF markers in PeCa-derived cells previously published by our group (40). The expression profile of Cell1 (healthy individual) was distinct from cells with fibroblast-like morphology (Cell4, Cell5, and Cell6, **Figure 5B**), which presented a high expression of CAF markers (**Figure 5A**). The expression levels of MMPs and collagen genes revealed two distinct clusters (all three CAF cell lines versus Cell1). Overall, MMPs were overexpressed (especially in Cell6), while collagens were down expressed in CAFs compared to Cell1 (**Figure 5C**). Corroborating our previous results (10), MMP1 was highly overexpressed in PeCa samples (Affymetrix dataset). This gene was also overexpressed in Cell6, while MMP7 and *MMP9* presented increased expression in Cell4 (**Figure 5C**).

We evaluated a compound that potentially inhibits the enzymatic activity of secreted proteins identified in our analysis, especially the MMPs (IPA software) (**Figure 5D**). Although a modest effect was observed when CAF cells were treated with a broad MMP inhibitor (GM6001), the concentration of ~10 μM of GM6001 promoted decreased cell viability in CAFs compared to Cell1 (**Figure 5E**).

## Discussion

In this study, we explored the transcriptome data of PeCa samples to evaluate the interplay between cells within the TME and its relevance to disease outcomes. We identified an enrichment of immune and stromal cells and an association with survival. A second and complementary approach was based on investigating targetable MMPs for TME modulation in PeCa.

We found that immune cells and CAFs play a critical role in the TME by expressing and potentially secreting inflammatory factors and ECM remodeling proteinases. We also verified that immune cell proportions were negatively correlated with CAFs in PeCa samples. Interestingly, patients with high CAF scores presented lower survival rates and an increased expression of MMPs and collagens. These results demonstrate that our strategy to profile and deconvolute bulk tumors brings new perspectives to understand the TME of PeCa better. These findings also provided the rationale to test, *in vitro*, the MMP inhibitor GM6001 on PeCa-derived CAFs. We observed a higher effect of this inhibitor in penile CAFs than in normal fibroblasts.

Extracellular components and inflammatory factors were the main class of upregulated secreted proteins found in our internal and validation PeCa datasets. We found 23 secretome genes shared in these two datasets. This small overlap could be explained by the different microarray platforms used or simply by the intrinsic heterogeneity found in cancer samples. However, enriched pathways and gene ontologies were mainly associated with extracellular matrix and immune response in both datasets, reinforcing their relevance to the disease despite the differences in the overlapping secretome. The immune-inflammatory system and matrix metalloproteases were previously demonstrated to be overrepresented in PeCa compared to normal penile tissues (10, 43). In oral carcinomas, high levels of pro-inflammatory cytokines affect the TME by increasing ECM degradation *via* MMPs during disease progression (44). Our findings suggest that interactions between inflammation and matrix remodeling have a crucial role in penile carcinogenesis and progression.

We found increased scores of B cells, macrophages, and DCs in PeCa compared to normal tissues. Moreover, we described that a subset of PeCa patients presented an immune hot phenotype (higher scores of CD8 in T cells, DC, and mast cells). These features are potentially associated with a better prognosis. Although the low number of our cases precluded statistical significance, B cells and DCs improve prognosis in cancer patients due to the antitumor activity and the potential to increase immunotherapy response (45–48). The immune hot score classification predicts a better prognosis in cancer patients (29). Altogether, the immune classification of PeCa could be used as a tool to predict the outcome and immunotherapy response, mainly because we also found a negative correlation between immune and CAF scores in PeCa. These results open new scenarios to test whether immunotherapy response could be enhanced using a combinatorial treatment with TME-modulating drugs.

The CAF score is increased in PeCa compared to normal samples, but we also found a subset of PeCa highly expressing CAF markers with lower overall survival (internal and validation sets). It has been established that CAF gene signatures can distinguish between low and high CAF tumors and predict patient survival (49, 50). The impact of CAFs on patients’ survival has been reported, and their inhibition has emerged as a promising anti-cancer therapy (51). However, the pharmaceutical inhibition of CAFs expressing the canonical marker *FAP* (fibroblast activation protein) has not been proven to be successful yet (51). CAFs contribute to an immunosuppressive TME and targeting CAFs, or their products have the potential to improve current immunotherapy approaches for cancer patients (42). Therefore, a comprehensive understanding of CAF markers is needed, aiming to design effective therapeutic strategies for PeCa.

We showed that PeCa presented a global alteration of MMPs and collagens, in which tumors with high CAF scores have an increased expression of collagens. *COL11A1* was associated with LN metastasis, corroborating with previous findings (52, 53), and a novel prognostic biomarker of PeCa. Collagens are the most abundant ECM component, increasing tumor tissue stiffness, among other features (54). MMPs are essential to degrade collagen during ECM remodeling (41). A previous study demonstrated that *MMP1* and *MMP12* presented increased expression in usual and mixed PeCa subtypes (10). Herein, we confirmed these alterations and found an increased expression of *MMP1* in tumors with high CAF scores. Epithelial cells express MMPs (55); thus, the inhibition of MMP must modulate the microenvironment and malignant epithelial cells. We showed that MMPs are highly expressed in PeCa cells and PeCa-derived CAFs. The implication of MMPs in tumor invasion and metastasis has prompted the development of strategies that promote MMP inhibition (56). A high expression of *MMP1* has been related to poor outcomes and shorter overall survival in PeCa (10). CAFs express MMPs that assist the immunosuppression of TME, counteracting CAFs that secrete MMPs, which have the potential to enhance the efficacy of immunotherapies (42). Therefore, MMP inhibition is a potential therapeutic strategy for PeCa, especially in combination with standard-of-care therapies.

PeCa–derived cell lines were previously described as reliable models to investigate the molecular mechanisms associated with carcinogenesis and treatment resistance and to develop effective treatment strategies (57). Targeting therapies enabled personalized approaches to improve the outcome of PeCa patients (7, 58). The genomic profiling of PeCas revealed the potential of Epidermal Growth Factor Receptor (EGFR) target therapy, in which tumors with *EGFR* amplification could be more sensitive (59). However, the number of preclinical studies in PeCa is still limited. Genetically engineered mouse models of PeCa were elegantly evaluated, showing that a combined target therapy and immunotherapy could be used in the treatment of PeCa patients (17).

In the present study, to better investigate the behavior of CAFs and the therapy response, we showed that the inhibition of MMPs using a broad-spectrum MMP inhibitor presented a modest effect in PeCa-derived CAFs (2 out of 3 cells) and no effect in normal fibroblasts. The slight alteration on cell viability was not surprising; the TME remodeling does not necessarily require CAFs to die but is often associated with the modulation of CAF functions (60). Cell4 had higher MMP inhibitor sensitivity than the other cells, which could be explained by the high expression of *MMP7* and *MMP9* (targets of GM6001). High levels of *MMP7* are associated with shorter survival in cancer patients, while the prognostic role of *MMP9* is controversial (61). Cell6 presented the higher expression levels of *MMP1* and *MMP12* (direct targets of GM6001) and showed a better response to MMP inhibition. Thus, the MMP modulation in the TMEs needs to be individually evaluated in different tumor types to design suitable MMP targeting therapies (61). Several clinical trials have tested MMP inhibitors during the last decades, and most of these studies failed due to the lack of efficacy and severe side effects (56). As more selective inhibitors of MMPs are now available, MMP targeting could be reconsidered for cancer therapy (56). Clinical trials with new MMP inhibitors and combined therapies should be undertaken to improve therapy efficacy for PeCa patients. Considering that extensive degradation of ECM proteins *via* MMPs promotes tumor invasion and metastasis (62), it is crucial to remodel the ECM for the most effective treatment. Therapeutic strategies targeting aberrant ECM components for cancer treatment can act as an adjuvant for conventional chemotherapy and immunotherapy (63).

Herein, we highlighted for the first time the role of CAFs and the interplay of cells within the TME in PeCa; however, this retrospective study also has limitations, including the small sample size. We overcome this limitation by validating the gene expression findings in an independent dataset. Additional studies are necessary to validate the computational prediction of cell proportions in the TME and its prognostic impact, such as single-cell RNA sequencing studies. Translational research for PeCa is still a challenge, but recent advances in PeCa patient–derived tumor xenografts demonstrate the potential of this model to design a personalized treatment considering the genomic and TME profiling (64).

## Conclusions

Our data highlight the interplay between cell types in the TME of penile carcinomas. We demonstrated the complexity of the TME and the association between immune cells and CAFs as a prognostic factor for PeCa patients. We found a global deregulation of collagens and MMPs and tested CAF cell lines using an MMP inhibitor, which proved the ability to modulate these cells. These findings pave the way for future studies to understand the impact of TME-modulating therapies in PeCa patients.

## Data Availability Statement

The datasets presented in this study can be found in online repositories. The names of the repository/repositories and accession number(s) can be found in the article/**Supplementary Material**.

## Ethics Statement

The studies involving human participants were reviewed and approved by the Human Research Ethics Committee from A.C.Camargo Cancer Center and Barretos Cancer Hospital, São Paulo, Brazil (Protocols 1884/14 and 1030/2015, respectively). The patients/participants provided their written informed consent to participate in this study.

## Author Contributions

SR: Concept, design, and supervision. HK, JJM, and JPS: Conduction of the experiments. SC, JSS, and FM: Bioinformatic analysis. SC, HK, and SR: Writing and editing the manuscript. AL, CS-N, and EF: Assistance with sample collection and clinical data. J-MD: Design and supervision of the drug assays. All authors: Data analysis and interpretation, reading, and approval of the final manuscript.

## Funding

This work was supported by the National Institute of Science and Technology in Oncogenomics (São Paulo Research Foundation – FAPESP: #2008/57887-9 and the National Council for Scientific and Technological Development – CNPq: #573589/08-9), and the Research Council of Lillebaelt Hospital, Denmark.

## Conflict of Interest

The authors declare that the research was conducted in the absence of any commercial or financial relationships that could be construed as a potential conflict of interest.

## Publisher’s Note

All claims expressed in this article are solely those of the authors and do not necessarily represent those of their affiliated organizations, or those of the publisher, the editors and the reviewers. Any product that may be evaluated in this article, or claim that may be made by its manufacturer, is not guaranteed or endorsed by the publisher.

## Abbreviations

PeCa, penile cancer; CAF, cancer-associated fibroblasts; TME, tumor microenvironment; ECM, extracellular matrix; MMPs, matrix metalloproteinases; HPV, human papillomavirus; DEGs, differentially expressed genes; HPA, human protein atlas; PPI, protein–protein interactions; FC, fold change; FDR, false discovery ratio.
