# Supplementary material for: Interplay Between Immune and Cancer-Associated Fibroblasts: A Path to Target Metalloproteinases in Penile Cancer
Source: Front Oncol. 2022 Jul 19;12:935093. doi: 10.3389/fonc.2022.935093 (PMC9343588; doi:10.3389/fonc.2022.935093)
Supplement: Supplementary file 1 [file DataSheet_1.docx]

Supplementary Material

# Supplementary Figure

**
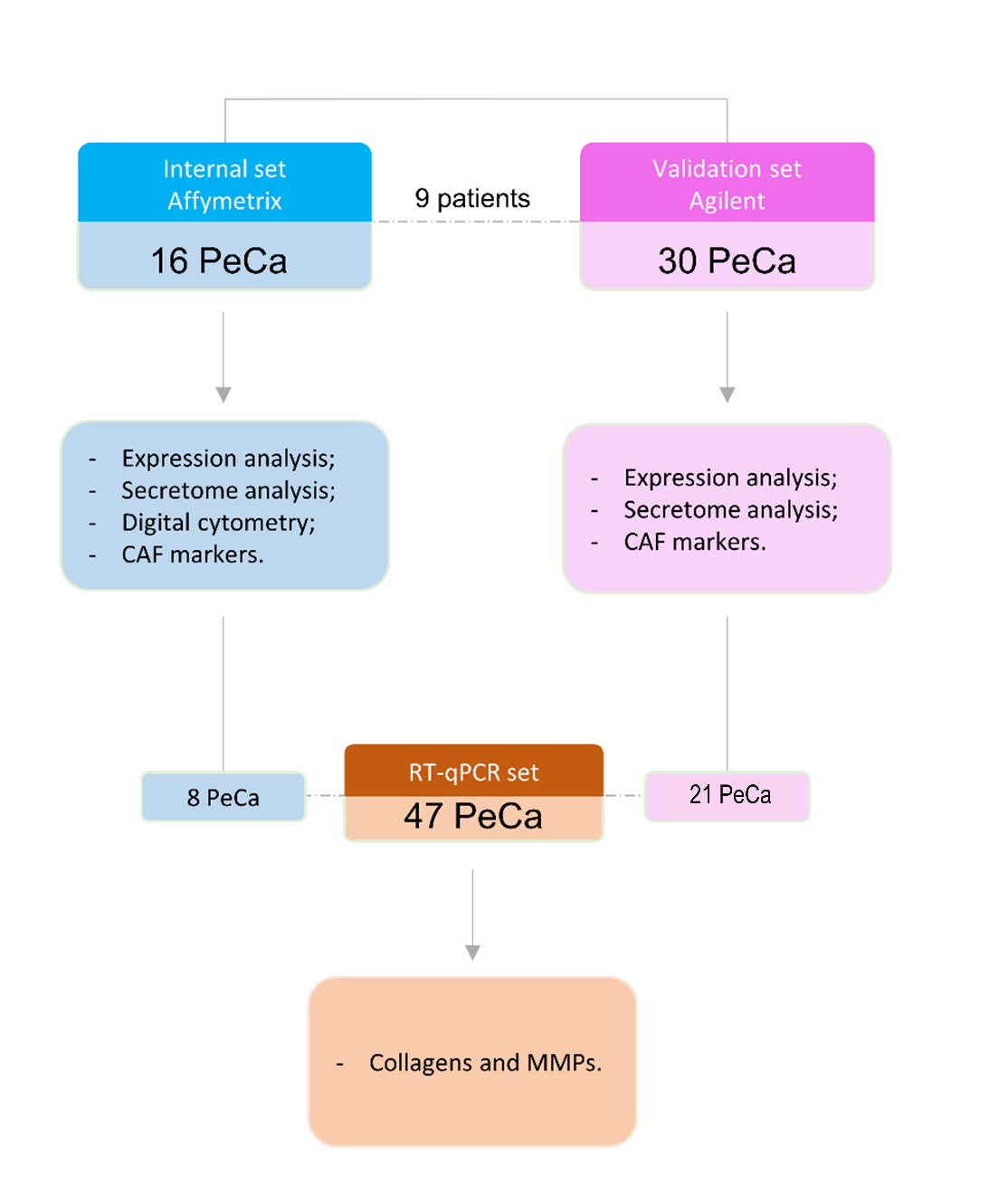
**

**Supplementary Figure 1.** Flowchart representing the strategies and analysis of our cohort of 63 penile cancer (PeCa) samples. Sixteen PeCa samples were included in the transcriptome analysis using GeneChip™ Human Transcriptome Array 2.0 (internal set), while 30 samples were included in the validation set (transcriptomic data using Agilent 44K platform). Nine cases were evaluated using both microarray platforms. RT-qPCR analysis was performed in 47 PeCa, in which 21 cases were included in the transcriptomic analyses. Boxes at the bottom of the figure indicate the subsequent analyses performed with each dataset of PeCa.

# Supplementary Tables

**Supplementary Table 1.** Clinical and histopathological characteristics of patients with usual penile squamous cell carcinomas analyzed using the microarrays platforms Agilent and Affymetrix*.

| **Characteristics** | **Internal set**  **Affymetrix, n=16 (%)** | **Validation set**  **Agilent, n=30 (%)** | **RT-qPCR set**  **n=47 (%)** |
| --- | --- | --- | --- |
| **Age (mean)** | 58.25 | 55 | 58 |
| **Tumor Grade** |  |  |  |
| G1 | 3 (18.75) | 8 (26.7) | 13 (27.6) |
| G2 | 7 (43.75) | 15 (50) | 24 (51.1) |
| G3 | 5 (31.25) | 7 (23.3) | 9 (19.2) |
| NA | 1 (6.25) | - | 1 (2.1) |
| **Tumor Stage** |  |  |  |
| I-II | 7 (43.75) | 16 (53.3) | 18 (38.3) |
| III-IV | 9 (56.25) | 12 (40) | 22 (46.8) |
| NA | - | 2 (6.7) | 7 (14.9) |
| **Smoking habit** |  |  |  |
| Yes | 5 (31.25) | 8 (26.7) | 14 (29.8) |
| No | 8 (50) | 20 (66.7) | 26 (55.3) |
| NA | 3 (18.75) | 2 (6.7) | 7 (14.9) |
| **Alcohol consumption** |  |  |  |
| Yes | 2 (12.5) | 5 (16.7) | 8 (17) |
| No | 10 (62.5) | 22 (73.3) | 31 (66) |
| NA | 4 (25) | 3 (10) | 8 (17) |
| **HPV status** |  |  |  |
| Yes | 4 (25) | 8 (26.7) | 13 (27.6) |
| No | 12 (75) | 22 (73.3) | 34 (72.3) |
| **Survival** |  |  |  |
| Alive | 10 (62.5) | 20 (66.7) | 26 (55.3) |
| Deceased | 6 (37.5) | 9 (30) | 11 (23.4) |
| NA |  | 1 (3.3) | 10 (21.3) |
| **cT stage** |  |  |  |
| 1-2 | 11 (68.75) | 17 (56.7) | 28 (59.6) |
| 3-4 | 5 (31.25) | 11 (36.7) | 16 (34) |
| NA | - | 1 (3.3) | 3 (6.4) |
| **cN stage** |  |  |  |
| Nx | 1 (6.25) | - | 1 (2.1) |
| N0 | 10 (62.5) | 20 (66.7) | 25 (53.2) |
| N1 | 2 (12.5) | 4 (13.3) | 5 (10.6) |
| N2+N3 | 3 (18.75) | 5 (16.7) | 13 (27.6) |
| NA | - | 1 (3.3) | 3 (6.4) |
| **Perineural invasion** |  |  |  |
| Yes | 6 (37.5) | 6 (20) | 12 (25.5) |
| No | 9 (56.25) | 23 (76.7) | 32 (68.1) |
| NA | 1 (6.25) | 1 (3.3) | 3 (6.4) |
| **Angiolymphatic invasion** |  |  |  |
| Yes | 5 (31.25) | 2 (6.7) | 6 (12.8) |
| No | 11 (68.75) | 24 (80) | 35 (74.5) |
| NA | - | 4 (13.3) | 6 (12.8) |
| **Recurrence** |  |  |  |
| Yes | 7 (43.75) | 9 (30) | 11 (23.4) |
| No | 9 (56.25) | 19 (63.3) | 23 (48.9) |
| NA |  | 2 (6.7) | 13 (27.6) |
| **Lymph node metastasis** | | |  |
| Yes | 3 (18.75) | 7 (23.3) | 17 (36.2) |
| No | 4 (25) | 9 (30) | 27 (47.4) |
| NA | 9 (56.25) | 14 (46.7) | 3 (6.4) |
| **Distant metastasis** |  |  |  |
| Yes | 3 (18.75) | 3 (10) | 2 (4.2) |
| No | 13 (81.25) | 25 (83.3) | 26 (55.3) |
| NA | - | 2 (6.7) | 19 (40.4) |
| **Type of surgery** |  |  |  |
| Total penectomy | 3 (18.75) | 5 (16.7) | 8 (17) |
| Partial penectomy | 10 (62.5) | 24 (80) | 36 (76.6) |
| NA | 3 (18.75) | 1 (3.3) | 3 (6.4) |

*Nine patients were analyzed by both Agilent and Affymetrix platforms. Twenty-one of 47 cases evaluated by RT-qPCR were included in the transcriptome analysis. cT: clinical tumor stage; cN: clinical node stage (AJCC 7th edition); HPV: human papillomavirus; NA: not available.

**Supplementary Table 2.** Primer sets used for RT-qPCR assays.

| **Gene** | **Sequence 5' > 3'** |
| --- | --- |
| *MMP1-F* | GGACCAACAATTTCAGAGAGTACA |
| *MMP1-R* | GGGTACATCAAAGCCCCGAT |
| *MMP3-F* | TGGAGATGCCCACTTTGATGA |
| *MMP3-R* | GTCAGGTCTGTGAGTGAGTGA |
| *MMP7-F* | CGGGAGGCATGAGTGAGCTA |
| *MMP7-R* | TTCCAGTTATAGGTAGGCCAAAGAA |
| *MMP9-F* | ATCCCCGGAGCGCCA |
| *MMP9-R* | ACGTAGCCCACTTGGTCC |
| *MMP10-F* | TCATGCCTACCCACCTGGA |
| *MMP10-R* | GGAATAAATTGGTGCCTGATGC |
| *MMP12-F* | CGTGGCATTCAGTCCCTGT |
| *MMP12-R* | AACACTGGTCTTTGGTCTCTCA |
| *MMP13-F* | GGACAAGTAGTTCCAAAGGCTAC |
| *MMP13-R* | AACATGAGTGCTCCAGGGTC |
| *COL1A2-F* | CCCCCTGGTATGACTGGTTTC |
| *COL1A2-R* | GGACCAACTGCACCTACTTCT |
| *COL3A1-F* | CTTCTCTCCAGCCGAGCTTC |
| *COL3A1-R* | TGTGTTTCGTGCAACCATCC |
| *COL4A1-F* | GCCAGACCATTCAGATCCCA |
| *COL4A1-R* | AGTAATTGCAGGTCCCACGG |
| *COL5A2-F* | CCCATCCAGTGTACCACGTA |
| *COL5A2-R* | CCATAAGCGAACTGAGACCC |
| *COL10A1-F* | TGGATCAGGCTTCAGGGAGT |
| *COL10A1-R* | CCATTTGACTCGGCATTGGG |
| *COL11A1-F* | GGACAAAAAGGCAGCAAGGG |
| *COL11A1-R* | GTTCACCATCACCTCCAGCA |
| *COL24A1-F* | CACGAGATAACCCAGCACGA |
| *COL24A1-R* | GGCATCTGAAGGACAGCCAA |

F: forward; R: reverse

**Supplementary Table 3**. Top pathways and ontology terms statistically significant enriched in penile cancer secretome (internal and validation sets).

| **INTERNAL SET** | | | | | |
| --- | --- | --- | --- | --- | --- |
|  | **Name** | **P-value** | **Adjusted p-value** | **Odds Ratio** | **Combined score** |
| **WikiPathway 2021 Human** | |  |  |  |  |
|  | miRNA targets in ECM and membrane receptors WP2911 | 5.12E-16 | 9.38E-14 | 109.42 | 3852.42 |
|  | Complement Activation WP545 | 1.66E-08 | 3.38E-07 | 47.96 | 859.07 |
| **KEGG 2021 Human** | |  |  |  |  |
|  | Protein digestion and absorption | 1.79E-16 | 2.58E-14 | 25.05 | 908.38 |
|  | Viral protein interaction with cytokine and cytokine receptor | 6.58E-14 | 4.74E-12 | 21.87 | 663.95 |
| **MSigDB Hallmark 2020** | |  |  |  |  |
|  | Epithelial Mesenchymal Transition | 7.70E-27 | 2.23E-25 | 24.07 | 1447.43 |
|  | Coagulation | 2.10E-14 | 3.04E-13 | 17.83 | 561.67 |
|  | IL-6/JAK/STAT3 Signaling | 3.40E-08 | 1.64E-07 | 15 | 257.96 |
|  | Allograft Rejection | 8.11E-11 | 7.84E-10 | 10.91 | 253.61 |
|  | Angiogenesis | 9.79E-06 | 0.00002838 | 20.48 | 236.22 |
|  | Complement | 8.81E-10 | 6.39E-09 | 10.06 | 209.81 |
|  | Interferon Gamma Response | 8.83E-09 | 5.12E-08 | 9.23 | 171.18 |
|  | Inflammatory Response | 8.14E-08 | 3.37E-07 | 8.42 | 137.42 |
|  | KRAS Signaling Up | 6.85E-07 | 0.000002482 | 7.62 | 108.22 |
|  | Apical Junction | 5.23E-06 | 0.00001685 | 6.85 | 83.29 |
| **Reactome 2016** | |  |  |  |  |
|  | Assembly of collagen fibrils and other multimeric structures Homo sapiens R-HSA-2022090 | 2.35E-21 | 3.72E-19 | 57.5 | 2731.13 |
|  | Extracellular matrix organization Homo sapiens R-HSA-1474244 | 2.13E-41 | 6.74E-39 | 28.7 | 2687.79 |
|  | Collagen biosynthesis and modifying enzymes Homo sapiens R-HSA-1650814 | 1.74E-18 | 1.37E-16 | 42.36 | 1732.28 |
|  | Collagen formation Homo sapiens R-HSA-1474290 | 5.78E-21 | 6.09E-19 | 37.15 | 1730.99 |
|  | Defective CHST3 causes SEDCJD Homo sapiens R-HSA-3595172 | 1.75E-05 | 0.0002334 | 94.15 | 1031.29 |
|  | Defective CHSY1 causes TPBS Homo sapiens R-HSA-3595177 | 1.75E-05 | 0.0002334 | 94.15 | 1031.29 |
|  | Defective CHST14 causes EDS, musculocontractural type Homo sapiens R-HSA-3595174 | 1.75E-05 | 0.0002334 | 94.15 | 1031.29 |
|  | ECM proteoglycans Homo sapiens R-HSA-3000178 | 5.74E-13 | 3.03E-11 | 32.99 | 929.89 |
|  | Collagen degradation Homo sapiens R-HSA-1442490 | 7.37E-10 | 3.33E-08 | 33.41 | 702.57 |
|  | Degradation of the extracellular matrix Homo sapiens R-HSA-1474228 | 1.51E-13 | 9.54E-12 | 20.44 | 603.48 |
| **GO Biological Process 2021** | |  |  |  |  |
|  | extracellular structure organization (GO:0043062) | 7.01E-42 | 4.56E-39 | 35.51 | 3365.06 |
|  | external encapsulating structure organization (GO:0045229) | 8.49E-42 | 4.56E-39 | 35.31 | 3339.21 |
|  | complement activation, classical pathway (GO:0006958) | 3.90E-09 | 2.85E-07 | 158.93 | 3077.38 |
|  | regulation of natural killer cell chemotaxis (GO:2000501) | 1.39E-07 | 0.000006026 | 168.46 | 2659.83 |
|  | extracellular matrix organization (GO:0030198) | 9.86E-42 | 4.56E-39 | 27.77 | 2621.66 |
| **GO Molecular Function 2021** | |  |  |  |  |
|  | platelet-derived growth factor binding (GO:0048407) | 6.17E-13 | 1.11E-10 | 225.4 | 6336.85 |
|  | CXCR3 chemokine receptor binding (GO:0048248) | 5.06E-06 | 0.0000828 | 188.33 | 2296.48 |
|  | CCR1 chemokine receptor binding (GO:0031726) | 5.06E-06 | 0.0000828 | 188.33 | 2296.48 |
|  | CCR5 chemokine receptor binding (GO:0031730) | 5.06E-06 | 0.0000828 | 188.33 | 2296.48 |
|  | vascular endothelial growth factor-activated receptor activity (GO:0005021) | 1.75E-05 | 0.000225 | 94.15 | 1031.29 |
| **GO Cellular Component 2021** | |  |  |  |  |
|  | collagen-containing extracellular matrix (GO:0062023) | 3.54E-54 | 3.43E-52 | 31.15 | 3834.36 |
|  | endolysosome lumen (GO:0036021) | 0.000634 | 0.005589 | 83.17 | 612.44 |
|  | endoplasmic reticulum lumen (GO:0005788) | 1.28E-15 | 6.22E-14 | 11.78 | 403.97 |
|  | basement membrane (GO:0005604) | 3.72E-06 | 0.00005148 | 16.66 | 208.25 |
|  | platelet alpha granule lumen (GO:0031093) | 1.11E-06 | 0.00001802 | 14.98 | 205.39 |
| **VALIDATION SET** | | | | | |
| **Wiki Pathways 2021 Human** | |  |  |  |  |
|  | Cytokines and Inflammatory Response WP530 | 3.4E-09 | 1.613E-07 | 40.06 | 781.22 |
|  | Matrix Metalloproteinases WP129 | 1.02E-08 | 2.685E-07 | 33.09 | 608.9 |
| **KEGG 2021 Human** | |  |  |  |  |
|  | Cytokine-cytokine receptor interaction | 9.83E-25 | 1.681E-22 | 15.15 | 837.45 |
|  | IL-17 signaling pathway | 5.13E-16 | 4.386E-14 | 23.4 | 823.75 |
| **MSigDB Hallmark 2020** | |  |  |  |  |
|  | TNF-alpha Signaling via NF-kB | 7.51E-12 | 2.93E-10 | 10.6 | 271.54 |
|  | IL-6/JAK/STAT3 Signaling | 1.35E-07 | 7.524E-07 | 12.65 | 200.08 |
|  | Coagulation | 7.71E-09 | 6.016E-08 | 10.59 | 197.86 |
|  | KRAS Signaling Up | 7.8E-10 | 1.52E-08 | 9.15 | 191.8 |
|  | Complement | 7.12E-09 | 6.016E-08 | 8.44 | 158.36 |
|  | Epithelial Mesenchymal Transition | 7.12E-09 | 6.016E-08 | 8.44 | 158.36 |
|  | Allograft Rejection | 6E-08 | 0.00000039 | 7.75 | 128.9 |
|  | Inflammatory Response | 4.66E-07 | 0.000002269 | 7.08 | 103.18 |
|  | p53 Pathway | 2.13E-05 | 0.00009237 | 5.77 | 62.05 |
|  | Estrogen Response Late | 0.000124 | 0.00044 | 5.14 | 46.2 |
| **Reactome 2016** | |  |  |  |  |
|  | Anchoring fibril formation Homo sapiens R-HSA-2214320 | 2.64E-07 | 0.00001026 | 142.76 | 2162.2 |
|  | Type I hemidesmosome assembly Homo sapiens R-HSA-446107 | 9.38E-07 | 0.000026 | 85.65 | 1188.75 |
|  | Chemokine receptors bind chemokines Homo sapiens R-HSA-380108 | 1.39E-13 | 2.688E-11 | 30.46 | 901.77 |
|  | Collagen degradation Homo sapiens R-HSA-1442490 | 2.63E-09 | 1.273E-07 | 28.2 | 557.22 |
|  | Degradation of the extracellular matrix Homo sapiens R-HSA-1474228 | 1.38E-12 | 1.784E-10 | 17.15 | 468.27 |
|  | Collagen formation Homo sapiens R-HSA-1474290 | 2.57E-11 | 2.497E-09 | 18.33 | 446.97 |
|  | Assembly of collagen fibrils and other multimeric structures Homo sapiens R-HSA-2022090 | 1.83E-09 | 1.014E-07 | 21.96 | 441.86 |
|  | Activation of Matrix Metalloproteinases Homo sapiens R-HSA-1592389 | 4.86E-07 | 0.0000145 | 24.95 | 362.7 |
|  | Extracellular matrix organization Homo sapiens R-HSA-1474244 | 3.22E-15 | 1.247E-12 | 10.42 | 347.68 |
|  | Non-integrin membrane-ECM interactions Homo sapiens R-HSA-3000171 | 1.23E-07 | 0.000005295 | 21.73 | 345.81 |
| **GO Biological Process 2021** | |  |  |  |  |
|  | chronic inflammatory response (GO:0002544) | 3.83E-08 | 0.000001883 | 428.32 | 7314.38 |
|  | positive regulation of natural killer cell chemotaxis (GO:2000503) | 3.83E-08 | 0.000001883 | 428.32 | 7314.38 |
|  | regulation of natural killer cell chemotaxis (GO:2000501) | 2.64E-07 | 0.00001028 | 142.76 | 2162.2 |
|  | granulocyte chemotaxis (GO:0071621) | 5.54E-21 | 5.168E-18 | 37.81 | 1763.58 |
|  | neutrophil chemotaxis (GO:0030593) | 9.36E-20 | 5.822E-17 | 36.85 | 1614.4 |
| **GO Molecular Function 2021** | |  |  |  |  |
|  | CCR1 chemokine receptor binding (GO:0031726) | 8.19E-06 | 0.00009556 | 159.75 | 1871.07 |
|  | CXCR3 chemokine receptor binding (GO:0048248) | 8.19E-06 | 0.00009556 | 159.75 | 1871.07 |
|  | cytokine activity (GO:0005125) | 5.02E-28 | 1.054E-25 | 24.75 | 1556.04 |
|  | chemokine activity (GO:0008009) | 1.05E-14 | 7.337E-13 | 39.44 | 1269.4 |
|  | RAGE receptor binding (GO:0050786) | 9.38E-07 | 0.00001641 | 85.65 | 1188.75 |
| **GO Cellular Component 2021** | |  |  |  |  |
|  | extrinsic component of external side of plasma membrane (GO:0031232) | 0.002396 | 0.01917 | 35.3 | 213.02 |
|  | Golgi lumen (GO:0005796) | 3.71E-08 | 0.000001261 | 12.24 | 209.45 |
|  | endoplasmic reticulum lumen (GO:0005788) | 3.02E-11 | 2.054E-09 | 8.21 | 198.92 |
|  | collagen-containing extracellular matrix (GO:0062023) | 1.24E-11 | 1.682E-09 | 7.16 | 179.79 |
|  | specific granule lumen (GO:0035580) | 2.61E-05 | 0.0004429 | 11.57 | 122.08 |
|  | |  |  |  |  |

Enrichment was calculated for each library using Enrichr tool (<https://maayanlab.cloud/Enrichr/>).

**Table S4**. Correlation parameters of immune scores and cancer associated fibroblasts (CAF) scores in penile carcinomas from the internal set (N=16).

|  |  | **CAF score**  **EPIC** | **CAF score**  **CIBERSORTx** |
| --- | --- | --- | --- |
| Mean Immune Score# | r | -0,3365 | -0,6031 |
|  | p-value | 0,2025 | 0,0134* |
| B cells | r | 0,1489 | -0,1254 |
|  | p-value | 0,5820 | 0,6435 |
| Plasma cells | r | 0,2954 | 0,3861 |
|  | p-value | 0,2666 | 0,1396 |
| T cells CD8 | r | -0,1813 | -0,4198 |
|  | p-value | 0,5017 | 0,1055 |
| T cells CD4 | r | 0,07542 | 0,3437 |
|  | p-value | 0,7813 | 0,1924 |
| NK cells | r | -0,2421 | -0,1063 |
|  | p-value | 0,3662 | 0,6952 |
| Macrophages | r | 0,1014 | 0,2028 |
|  | p- value | 0,7087 | 0,4513 |
| Monocytes | r | -0,4216 | -0,2666 |
|  | p-value | 0,1039 | 0,3183 |
| Dendritic cells | r | -0,02423 | -0,2570 |
|  | p-value | 0,9290 | 0,3367 |
| Mast cells | r | 0,1206 | -0,02855 |
|  | p- value | 0,6563 | 0,9164 |
| Eosinophils | r | -0,1624 | 0,1383 |
|  | p- value | 0,5479 | 0,6094 |
| Neutrophils | r | -0,01907 | 0,07886 |
|  | p- value | 0,9441 | 0,7716 |

#Mean score of immune cells generated by CIBERSORTx in each sample. *Significant Pearson correlation.
